# Supplementary material for: Exploring CAR T-Cell Dynamics: Balancing Potent Cytotoxicity and Controlled Inflammation in CAR T-Cells Derived from Systemic Sclerosis and Myositis Patients
Source: Int J Mol Sci. 2025 Jan 8;26(2):467. doi: 10.3390/ijms26020467 (PMC11765450; doi:10.3390/ijms26020467)
Supplement: Supplementary file 1 [file ijms-26-00467-s001.zip › ijms-3369539-supplementary.pdf]

Supplementary Materials:

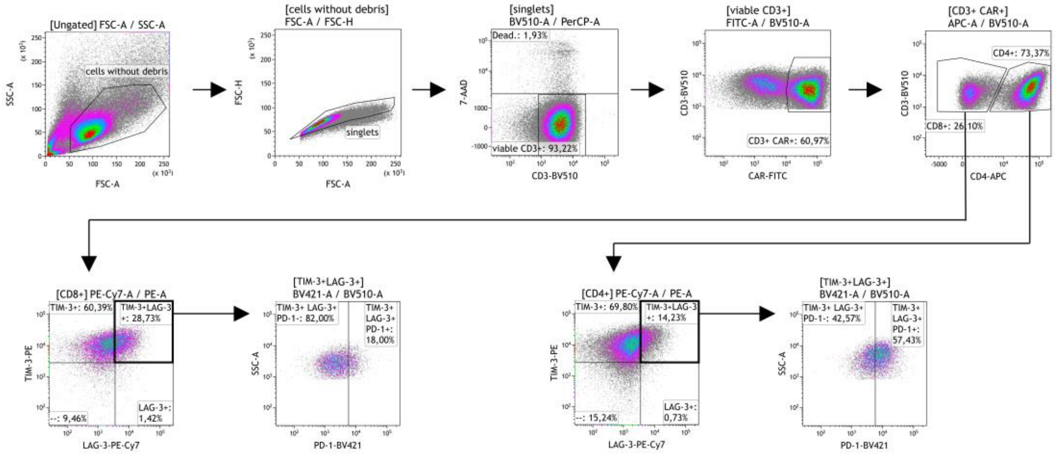

Figure S1. Gating strategy for exhaustion phenotyping of patient-derived CAR T cells. Representative dot plots of flow cytometric analysis of patient-derived CD3⁺ CAR T cells for exhaustion markers TIM-3, LAG-3, PD-1. Cell debris was excluded by FSC-A/SSC-A, doublets were excluded by FSC-H/FSC-A, and CAR T cells were gated by CD3⁺ and CAR⁺ and further subdivided into CD4⁺ and CD8⁺ T cells. CD4 and CD8 CAR T cells were analyzed for TIM-3 and LAG-3 co-expression. TIM-3⁺/LAG-3⁺ cells were further subdivided into PD-1⁺ CAR T cells and PD-1⁻ CAR T cells.

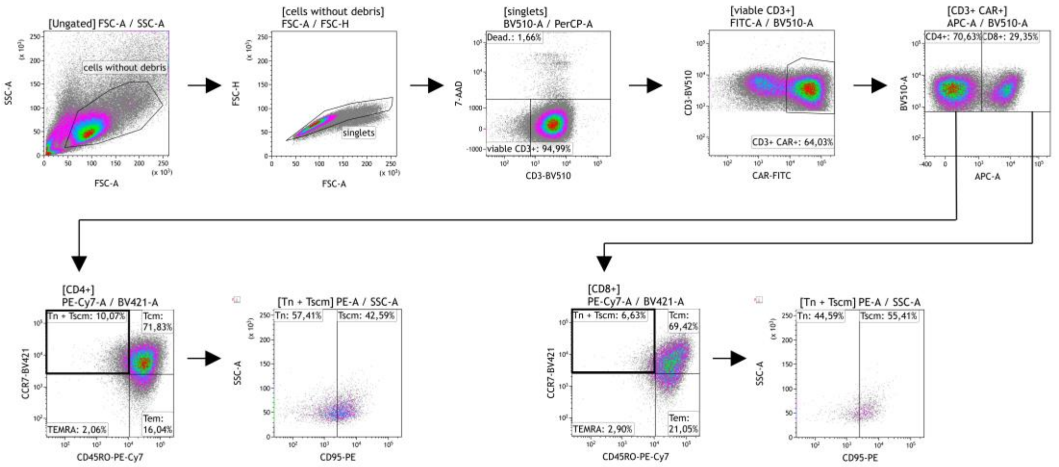

Figure S2. Gating strategy for memory phenotyping of patient-derived CAR-T cells. Representative dot plots of flow cytometric analysis of T cells for CCR7, CD45RO and CD95 distinguishing naïve T cells (Tn, CCR7⁺/CD45RO⁻/CD95⁻), stem cell-like T cells (Tscm, CCR7⁺/CD45RO⁻/CD95⁺), central memory T cells (Tcm, CCR7⁺/CD45RO⁺), effector memory T cells (Tem, CCR7⁻/CD45RO⁺) and Tem re-expressing CD45RA (TEMRA, CCR7⁻/CD45RO⁻). Cell debris was excluded by FSC-A/SSC-A, doublets were excluded by FSC-H/FSC-A, and viable T cells were gated by CD3⁺ and 7-AAD⁻ and further subdivided into CAR⁺ T cells. CAR⁺ cells were subdivided into CD4 and CD8 T cells and analyzed for CD45RO, CCR7 expression. CCR7⁺/CD45RO⁻ cells were further subdivided into CD95⁺ Tscm and CD95⁻ Tn cells.

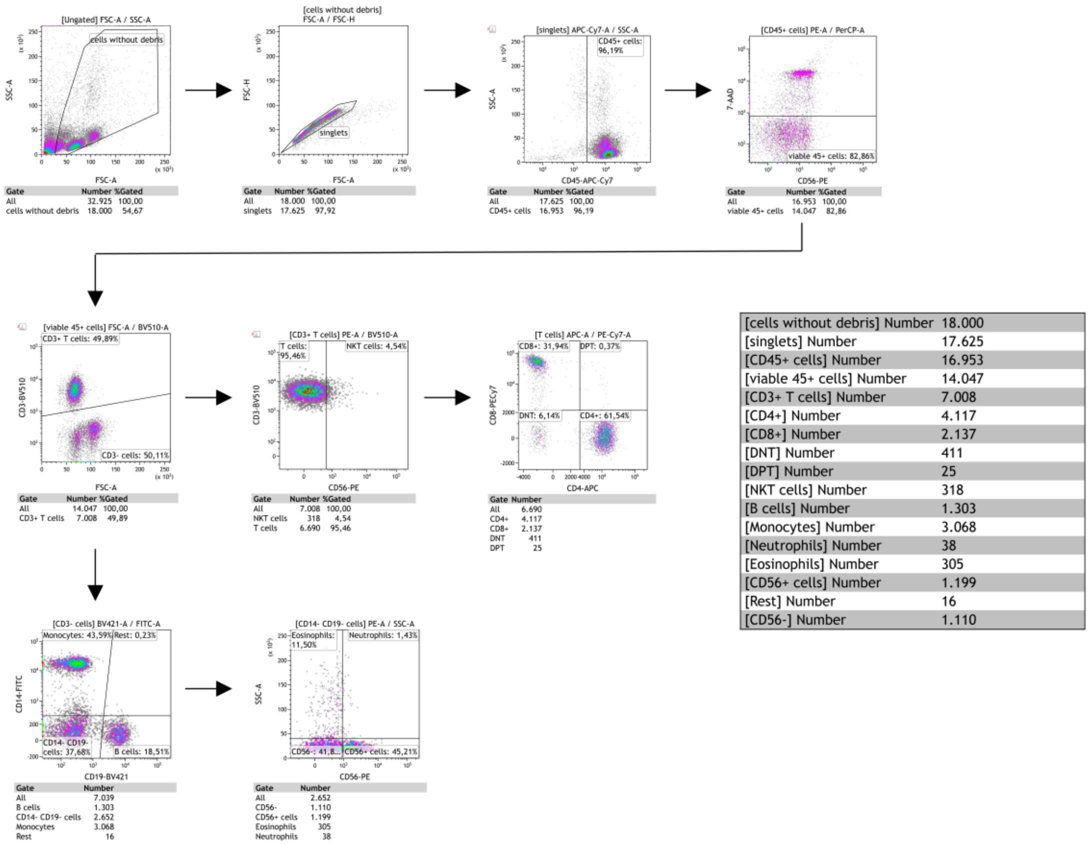

[cells without debris] Number	18.000
[singlets] Number	17.625
[CD45+ cells] Number	16.953
[viable 45+ cells] Number	14.047
[CD3+ T cells] Number	7.008
[CD4+] Number	4.117
[CD8+] Number	2.137
[DNT] Number	411
[DPT] Number	25
[NKT cells] Number	318
[B cells] Number	1.303
[Monocytes] Number	3.068
[Neutrophils] Number	38
[Eosinophils] Number	305
[CD56+ cells] Number	1.199
[Rest] Number	16
[CD56-] Number	1.110

Figure S3. Gating strategy for immunophenotyping of patient-derived PBMCs. Representative dot plots of flow cytometric analysis of patient-derived PBMCs. Cell debris was excluded by FSC-A/SSC-A, doublets were excluded by FSC-H/FSC-A and viable CD45+ cells were gated by D45+ and 7-AAD$^-$, followed by further subdivision into distinct immune cell subsets. These subsets include: CD3$^+$ CD4$^+$ T cells, CD3$^+$ CD8$^+$ T cells; double positive T cells (DPT cells, CD3$^+$ CD4$^+$ CD8$^+$), double negative T cells (DNT cells, CD3$^+$ CD4$^-$ CD8$^-$ T cells), natural killer T cells (NKT cells, CD3$^+$ CD56$^+$), monocytes (CD3$^-$ CD14$^+$), B cells (CD3$^-$ CD19$^+$) natural killer cells (NK cells, CD3$^-$ CD14$^-$ CD19$^-$ CD56$^+$ cells), eosinophil and basophil Granulocytes (SSC$^+$ CD3$^-$ CD14$^-$ CD19$^-$) and NK cells (SSC$^-$ CD3$^-$ CD14$^-$ CD19$^-$ CD56$^+$).

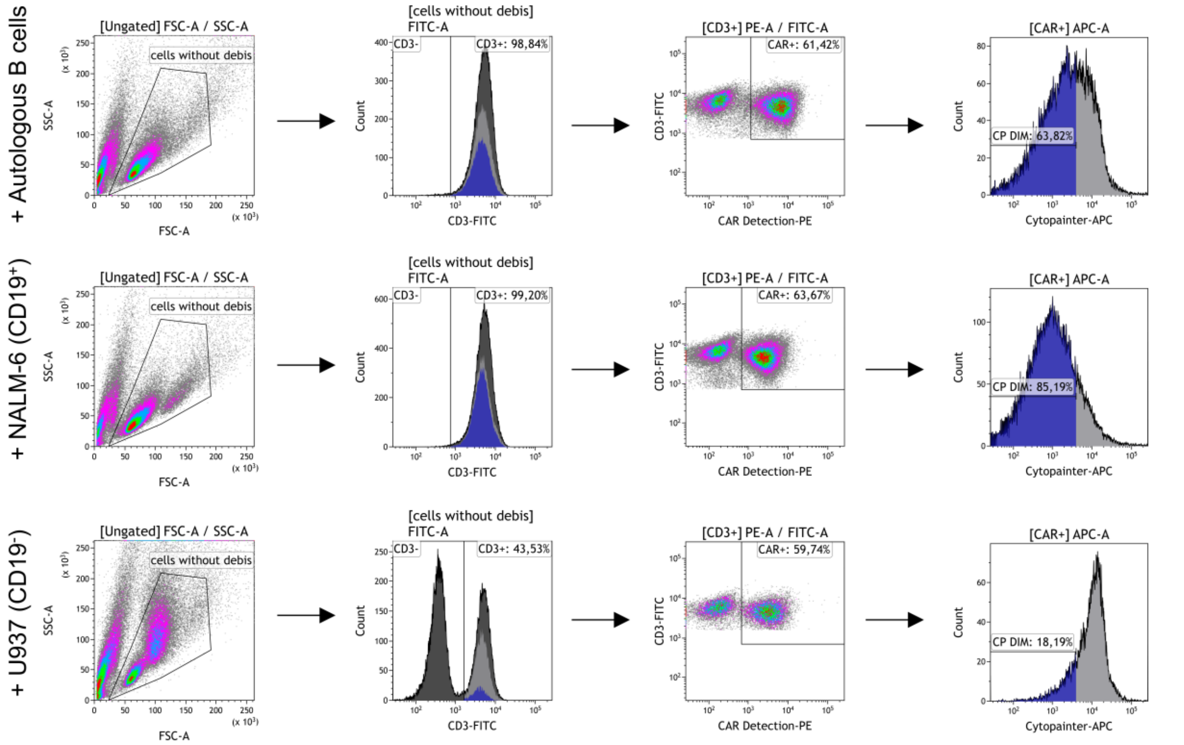

Figure S4. Gating strategy illustrating proliferation of patient-derived CAR-T cells. Representative flow cytometric plots of Cytopainter Deep Red dilution to track T cell proliferation at day 4 after target cell contact. Cell debris was excluded by FSC-A/SSC-A; CAR T cells were gated by CD3$^+$ CAR$^+$ and proliferating CAR T cells were gated by Cytopainter Deep Red dilution (CP Dim).

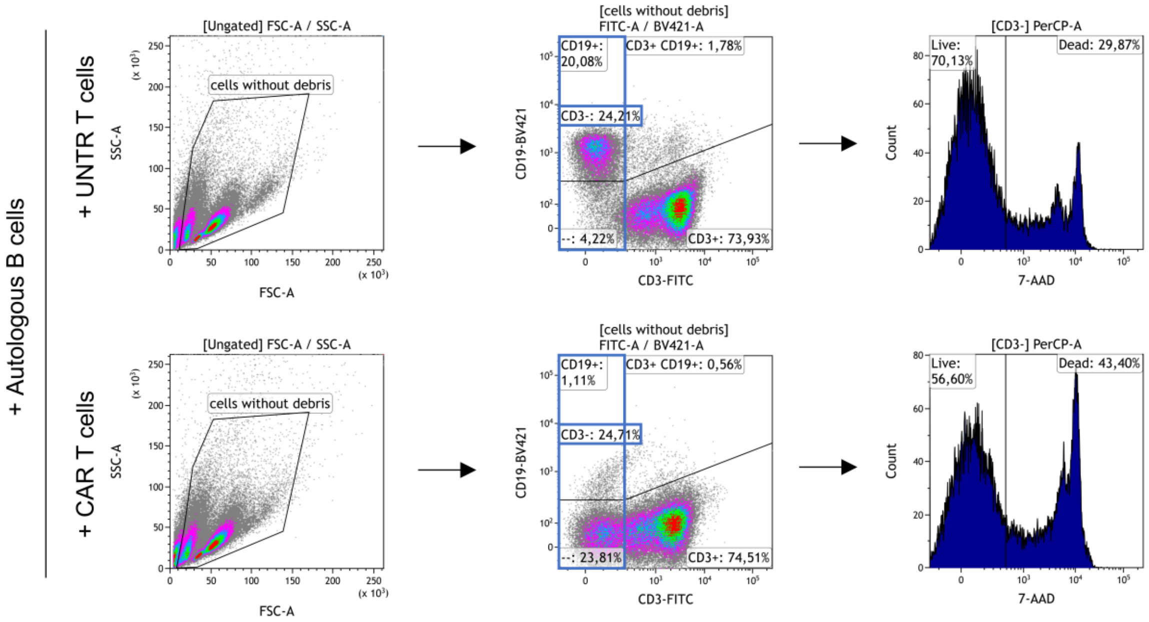

Figure S5. Gating strategy demonstrating cytotoxicity of patient-derived CAR-T cells. Representative flow cytometric plots of CAR T cell cytolysis measured by 7-AAD$^+$ CD3$^-$ target cells. Cell debris was excluded by FSC-A/SSC-A; T cells were gated as CD3$^+$ and target cells were gated as CD3$^-$ due to the interference caused by the targeting of CD19 by the CAR T cells. Target cells were subdivided in live cells by CD3$^-$ 7-AAD$^-$ and dead cells by CD3$^-$ and 7-AAD$^+$.
